# Supplementary material for: Effect of Polycan, a β-Glucan from Aureobasidium pullulans SM-2001, on Inflammatory Response and Intestinal Barrier Function in DSS-Induced Ulcerative Colitis
Source: Int J Mol Sci. 2023 Sep 30;24(19):14773. doi: 10.3390/ijms241914773 (PMC10572787; doi:10.3390/ijms241914773)
Supplement: Supplementary file 1 [file ijms-24-14773-s001.zip › ijms-2627488-supplementary.pdf]

Supplement data Table S1. Specific primer sequences for PCR

Mouse gene	Forward primer sequence (5’-3’)	Reverse Primer Sequence (5’-3’)
IL-1β	5’-CAGGATGAGGACATGAGC-3’	5’-CTCTGCAGACTCAAACTCCA-3’
TNF-α	5’-TCTGTCTACTGAACTTCGGGGTGATCGGTCC-3’	5’-GTATGAGATAGCAAATCGGCTGACGGTGTGGG-3’
IL-6	5’-TCCAGTTGCCTTCTTGGGAC-3’	5’-GTGTAATTAAGCCTCCGACTTG-3’
Caspase-3	5′-GGC TTG CCA GAA GAT ACC GGT-3′	5′-GCA TAA ATT CTA GCT TGT GCG CGT A-3′
PARP	5’-CCAGCGCAGCTCAGAGAAGCCA-3′	5’-CATGTTCGATGGGAAAGTCCC-3′
Bax	5’-CTACAGGGTTTCATCCAG -3′	5’-CCAGTTCATCTCCAATTCG-3′
Bcl-2	5’-GTGGATGACTGAGTACCT -3′	5’-CCAGGAGAAATCAAACAGAG-3′
F4/80	5′-TTTCCTCGCCTGCTTCTTC-3′	5′-CCCCGTCTCTGTATTCAACC-3′
Zo-1	5′-AGGACACCAAAGCATGTGAG-3′	5′-GGCATTCCTGCTGGTTACA-3′
GAPDH	5’-AACGACCCCTTCATTGAC-3’	5’-TCCACGACATACTCAGCAC-3’

Supplement data Table S2. Intestinal microbiota distribution at phylum level

Phylum	CTL	DSS	DSS+ 5-ASA	DSS+ Polycan 250	DSS+ Polycan 500
Bacteroidetes	63.36%	43.87%	60.82%	60.25%	41.80%
Firmicutes	34.99%	40.44%	26.06%	22.90%	19.37%
Proteobacteria	0.85%	11.89%	9.21%	10.84%	34.45%
Deferribacteres	0.34%	1.92%	1.99%	4.06%	1.58%
Actinobacteria	0.30%	0.12%	0.09%	0.75%	2.65%
Verrucomicrobia	0.00%	0.28%	0.01%	0.01%	0.00%
Lentisphaerae	0.00%	0.00%	0.00%	0.00%	0.00%
Tenericutes	0.00%	0.12%	0.00%	0.03%	0.00%
_Unclassified	0.17%	1.36%	1.82%	1.17%	0.14%
